# Supplementary material for: The integrated molecular and histological analysis defines subtypes of esophageal squamous cell carcinoma
Source: Nat Commun. 2024 Oct 18;15:8988. doi: 10.1038/s41467-024-53164-x (PMC11487165; doi:10.1038/s41467-024-53164-x)

Supplementary Figure 1. Transcriptomic clusters identified by non-negative matrix factorization (NMF). a, Consensus matrices of 120 ESCC tumour samples, computed for k = 2 to k = 7. Cophenetic correlation coefficient plot reveals peak cluster stability for k = 4. **b**, The subtype gene signatures were validated in three independent ESCC cohorts. The three cohorts are GSE53625 (n = 179, Li et al., 2014), GSE47404 (n = 71, Sawada et al., 2016) and TCGA ESCC cohort (n = 90). Source data are provided as a Source Data file.

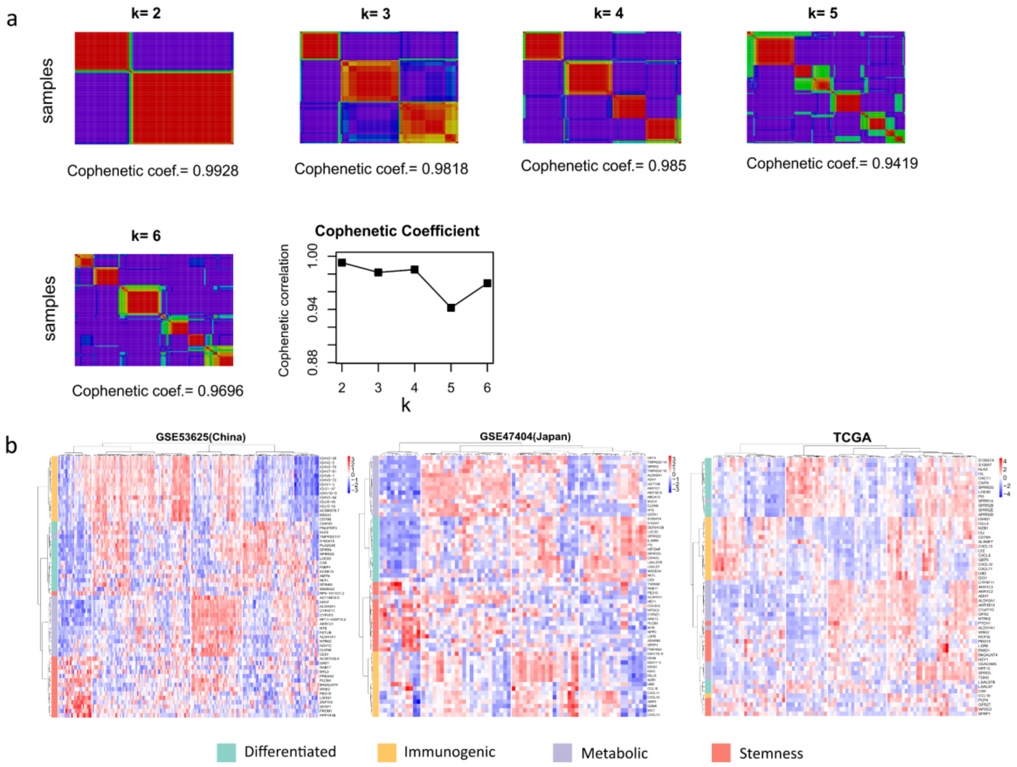

Supplementary Figure 2. The gene expression profile of differentially expressed transcription factors across the four subtypes. The full transcription factor list was obtained and fully reviewed by Lambert et al. Cell 2018, PMID: 29425488. Source data are provided as a Source Data file.

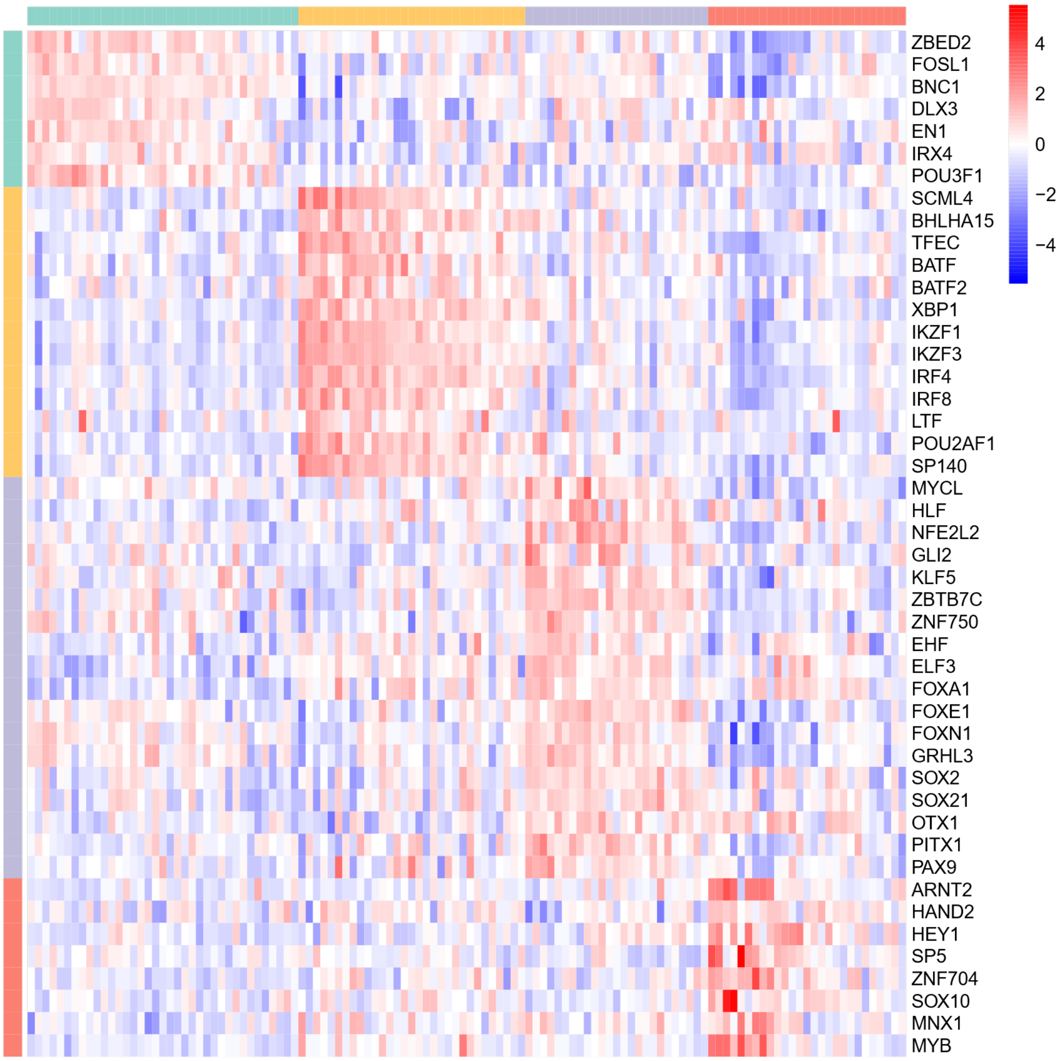

Supplementary Figure 3. Single-cell transcriptomes derived from 60 ESCC patients showed differential expression of the featured genes of the four subtypes. a, UMAP plot of 208,659 cells, coloured by cell types. b, MS4A1, CD79A, CXCL9 and MZB1 highly expressed in the Immunogenic subtype are expressed in immune (B and myeloid) cells and fibroblast cells. Although the feature genes for Differentiated(**c**), Metabolic(**d**) and Stemness(**e**) subtypes were predominately expressed in epithelia cells, they showed differential expression among the epithelia subpopulations.

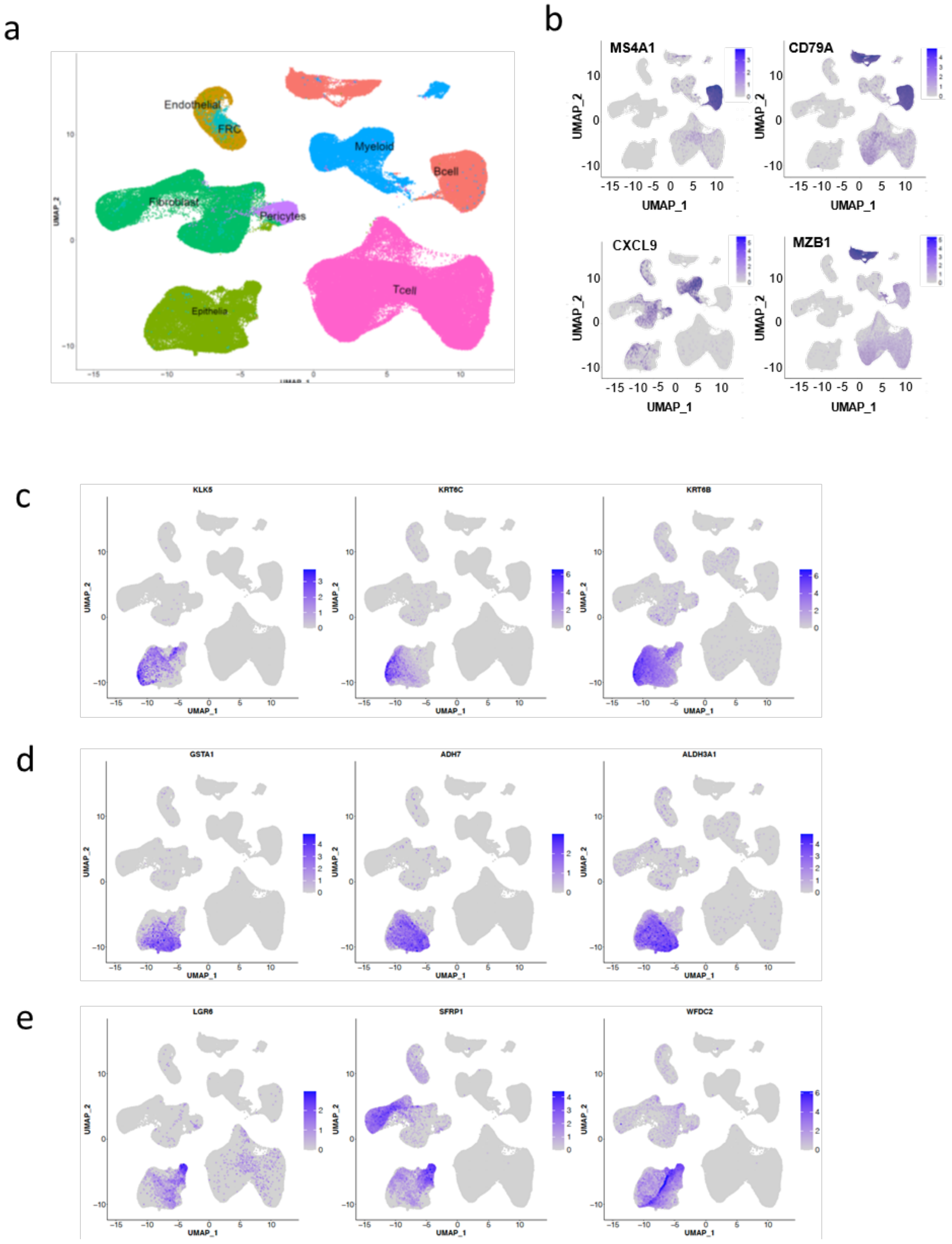

Supplementary Figure 4. Investigation of the heterogeneity of ESCC epithelial cells. a, 10 NMF clusters were identified using ESCC epithelial cells from Zhang et al., 2021. Downsampled heatmap is shown. Selected marker genes of each NMF clusters were listed, and their corresponding transcriptomic subtypes and expression programs of Zhang et al., 2021 were identified. b, Number and percentage of epithelial cells in each NMF cluster, as well as their corresponding transcriptomic subtypes and expression programs.

a

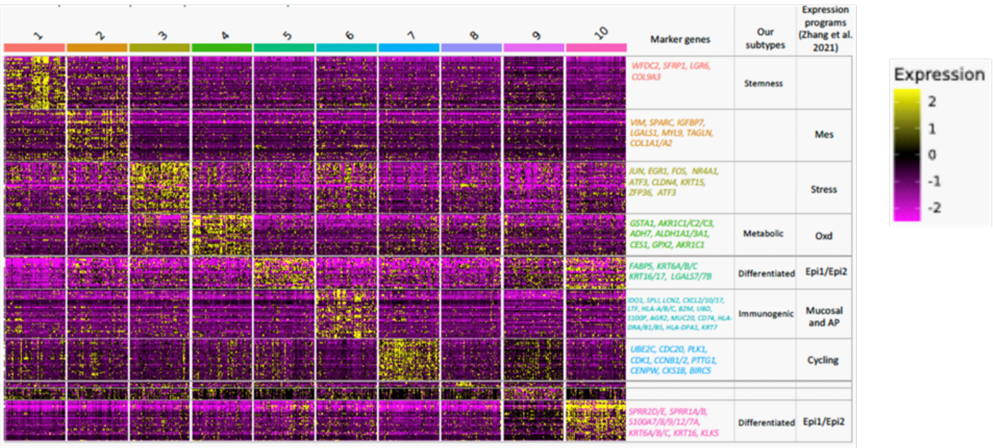

b

NMF of epithelial cells	# of cells	Percentage of total epi cells	Expression programs (Zhang et al. 2021)	Our transcriptomic subtypes
cluster1	5858	13%		Stemness
cluster2	3597	8%	Mes	
cluster3	5843	13%	Stress	
cluster4	5239	12%	Oxd	Metabolic
cluster5	5035	11%	Epi1/Epi2	Differentiated
cluster6	3298	7%	Mucosal and AP	Immunogenic
cluster7	4518	10%	Cycling	
cluster8	4522	10%		
cluster9	4259	10%		
cluster10	2427	5%	Epi1/Epi2	Differentiated

Supplementary Figure 5. *In vivo* experiments of SFRP1. a, Representative images of immunohistochemistry staining of SFRP1 negative or positive in human ESCC tumours. **b**, SFRP1 expression level in a panel of human ESCC cell lines were evaluated by Western blot assay. Cell proliferation in SFRP1 overexpressed KYSE-70 (**c**), KYSE-140 (**d**) and SFRP1-downregulated KYSE-520 (**e**), KYSE-450 (**f**) cells were validated using the Incucyte system, phase object confluence was compared using Two-way ANOVA. The matched Western Blot data indicates the SFRP1 expression level in different cells, also shown as barplots indicating, **g,** 20-40% knockdown efficiency in KYSE-520 cells, and **h,** 50% knockdown efficiency in KYSE-450 cells. Xenograft nude mouse model of *in vivo* tumourigenesis was performed to evaluate the SFPR1 effect on ESCC cell growth after overexpression(**i**) (n = 6) or knock-down(**j**) (n = 4, two mice had no tumour formation) of SFRP1. The resected tumour photos taken at 30 days after injection of the different tumour cells are presented, tumour size was measured and analysed with matched samples t-test. Data are presented as mean values + standard deviation. Source data are provided as a Source Data file.

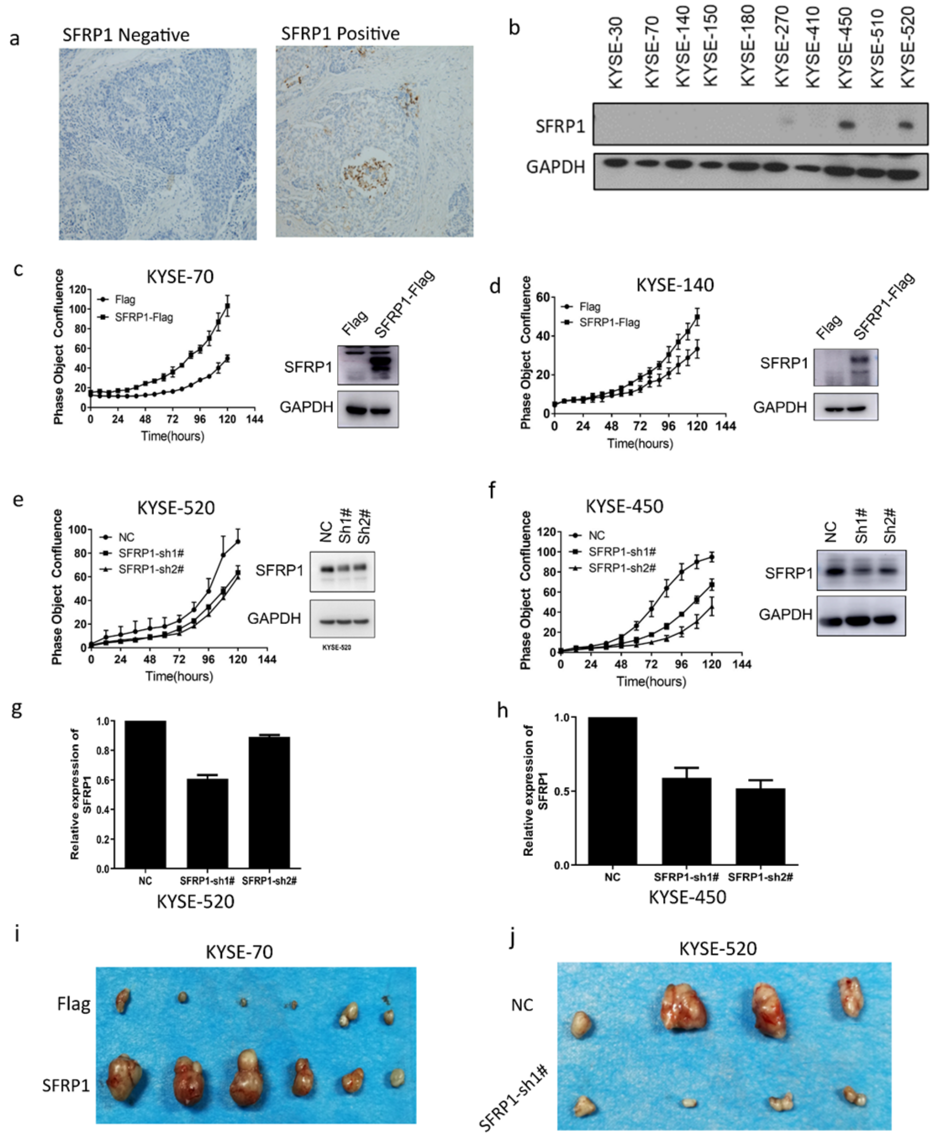

Supplementary Figure 6. Deep-learning of histology H&E slides to infer the gene expression classification. a, The workflow of the deep-learning feature extraction and inference of the transcriptomic subtypes. **b**, The comparison of subtype specific histology feature scores extracted from the deep-learning model across the four transcriptomic subtypes. The whiskers extending to a maximum of 1.5 times the interquartile range beyond the box. **c**, Representative histopathology images for the four subtypes are shown. A deep-learning model was developed to extract and compare subtype specific histological features based on histology slides. Top three representative tiles with the highest subtype specific histopathological feature scores were selected for each subtype. These features clearly discriminate the molecular subtypes. Source data are provided as a Source Data file.

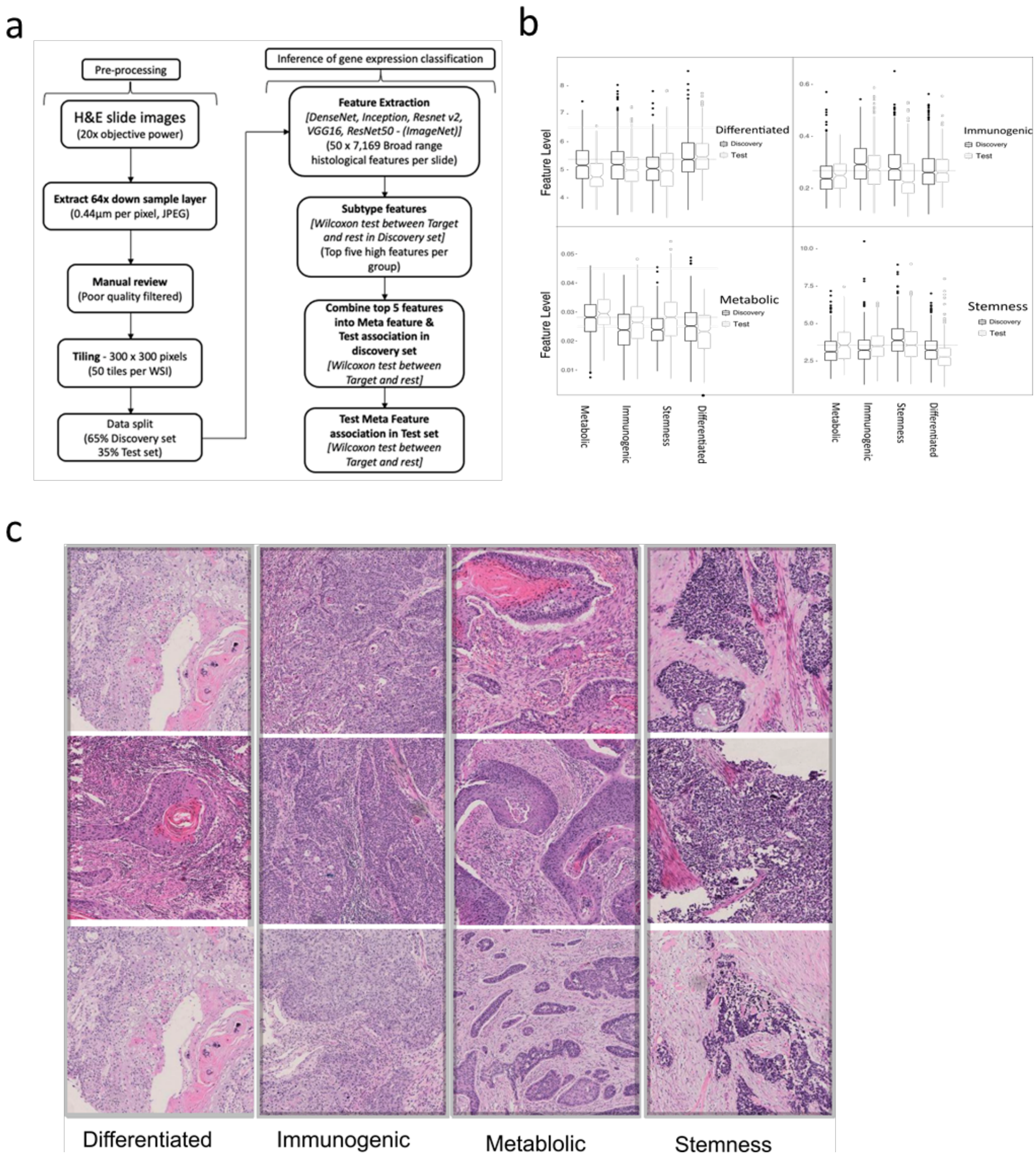

Supplementary Figure 7. Benchmarking published in silico immune deconvolution methods. a-b, The scatter plot between the in silico estimates (y axis) and histopathology estimates (x axis) for CD8+ (a) and CD4+ (b) T cells is shown, across selected published immune signatures, Danaher et al., Davoli et al., MCP-counter, Rooney et al., Timer and xCell. Danaher et al., and Rooney et al. signatures performed the best for CD8+ estimates, while Davoli et al. gave the best performance in estimating CD4+ cells, and this CD4+ signature was subsequently included into our Danaher et al., estimates for CD4+ T cells. c-d, The expression of the genes used in each of the immune signature definitions is correlated against tumour purity (c) and local copy number depth ratio (d). The Pearson's correlation coefficients were used for the plot, and the median value for each signature was shown by a solid horizontal red line. At the bottom, for each method, the percentage of genes that showed significantly negative correlations with tumour purity ($p < 0.05$) for (c) and no significant correlations with local copy number profile ($p > 0.05$) for (d) is shown. Source data are provided as a Source Data file.

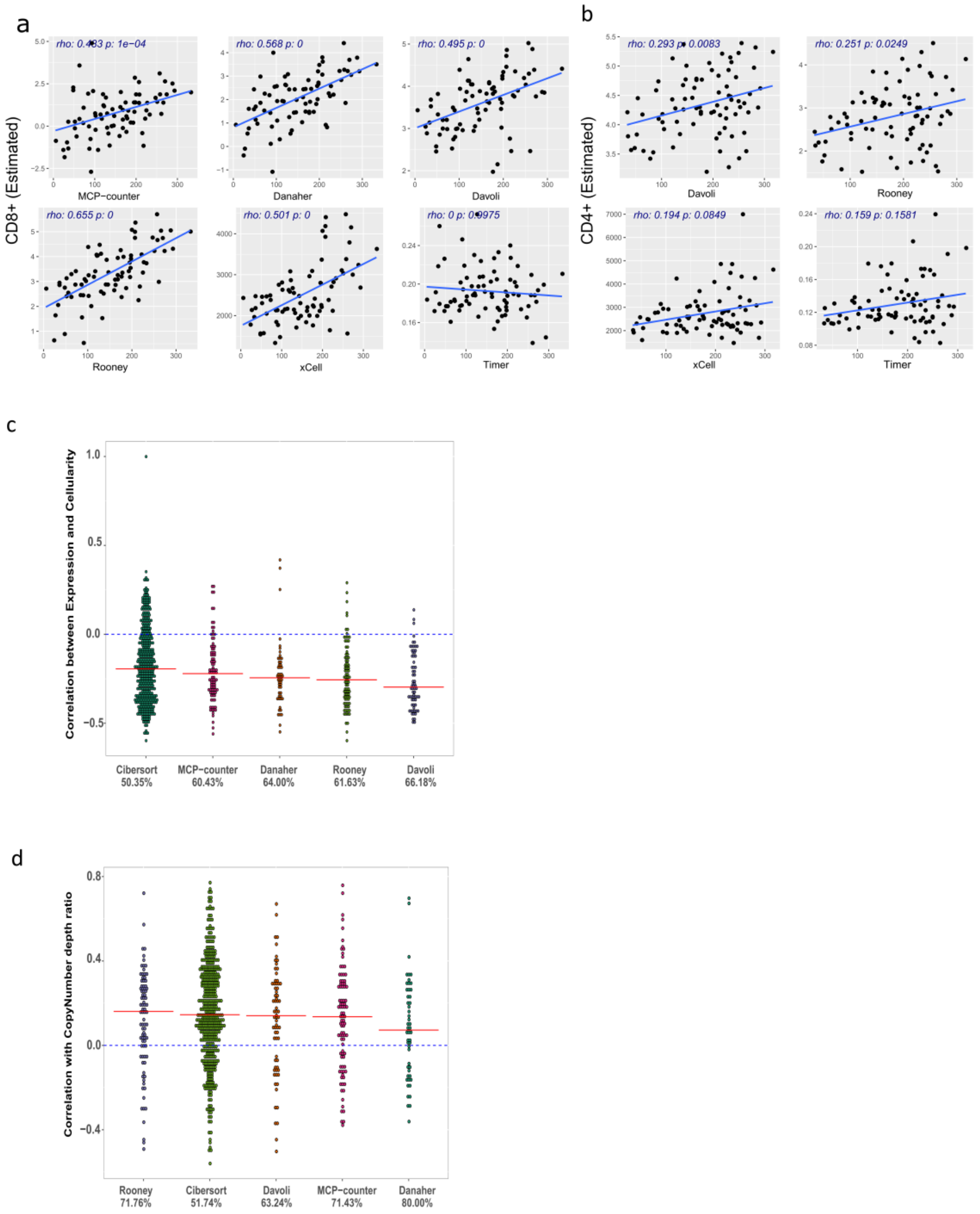

Supplementary Figure 8. The expression CD160 and XCL1 in our cohort. a, Gene expression of *CD160*, *NKG7* and *KLRC1* across the three immune clusters. The whiskers extending to a maximum of 1.5 times the interquartile range beyond the box，the significance derived from the Wilcoxon test is shown, * $p < 0.05$. **b,** The correlation between XCL1/2 expression and tumour cellularity derived from WES data. **c**, Two cases of ESCC were stained for XCL1 / LGR6 and CD160 / LGR6 co-expression, respectively. **d**, IHC staining of CD160 for two cases in esophageal submucosal gland. **E**, Survival plot of *CD160* expression in melanoma and renal cancer, showing high *CD160* expression is associated with poorer overall survival. The survival data here were from the Human Protein Atlas. Source data are provided as a Source Data file.

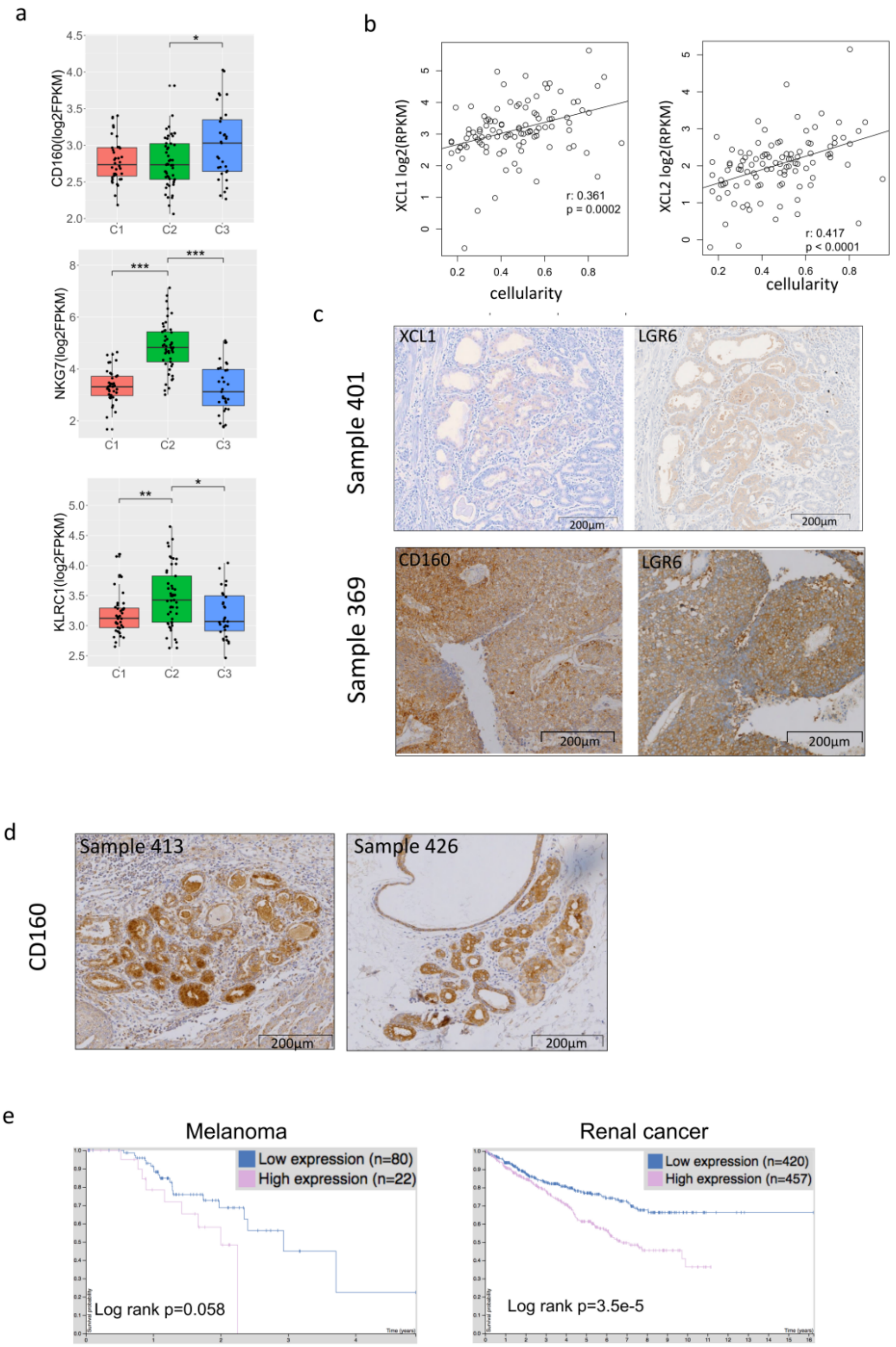

Supplementary Figure 9. The specificity and effectiveness of antibodies for CD3, CD4, CD8, CD56 and CD160 were validated by IHC assay using PBS and normal IgG as negative control.

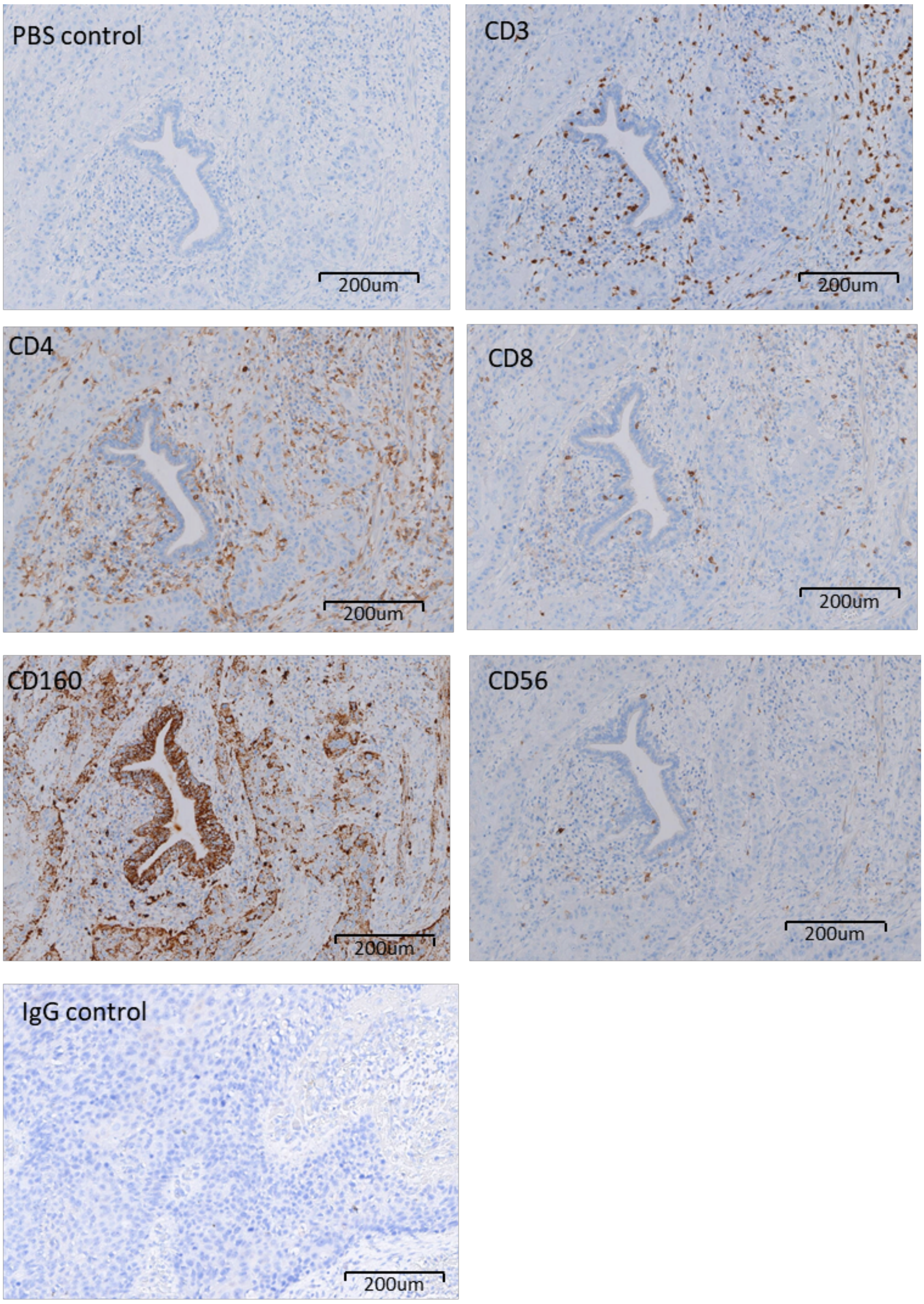

Supplementary Figure 10. The expression profile of CD4, CD8 and CD56 determined using IHC staining. a, Immunogenic samples showed higher CD4, CD8 and CD56 expression. Statistical significance was derived from two-sided Wilcoxon rank sum test is shown, * *p*<0.05, ** *p*<0.01, *** *p*<0.001. **b,** CD4 IHC expression showed little difference among three immune clusters. **c**, The intra-tumour heterogeneity, measured as the Shannon density, are negatively correlated with CD8 and CD56 IHC measurement, yet not significantly with CD4 as Pearson correlation and *p* values indicated. In a and b the box bounds the interquartile range divided by the median, with the whiskers extending to a maximum of 1.5 times the interquartile range beyond the box. Source data are provided as a Source Data file.

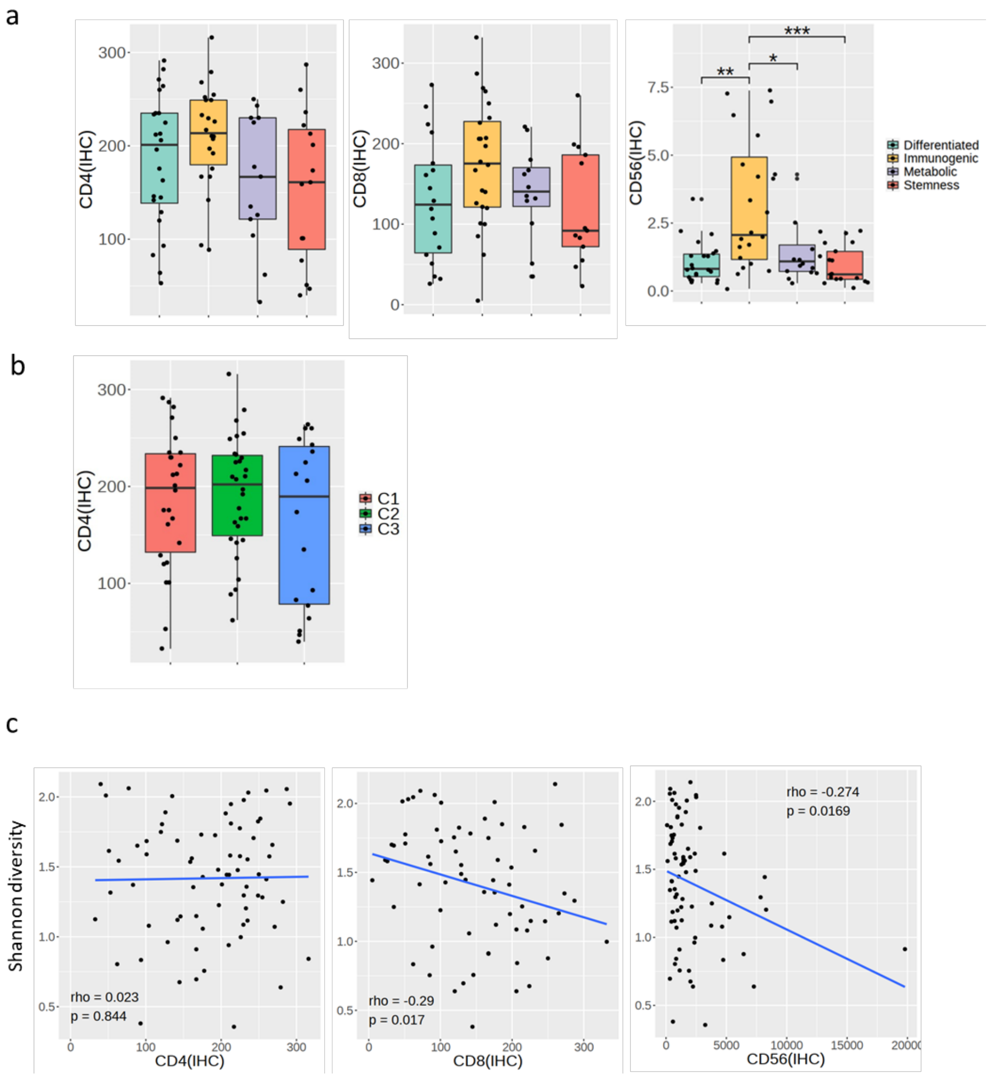

Supplementary Figure 11. Relative expression of XCL1, LGR6, CD56 and CD160 in paired adjacent normal and ESCC tissues from Sample 333 were analysed with IHC assay (a). b, IHC staining of XCL1, LGR6 and CD160 in serial sections of tumour tissue of Sample 341 was also shown.

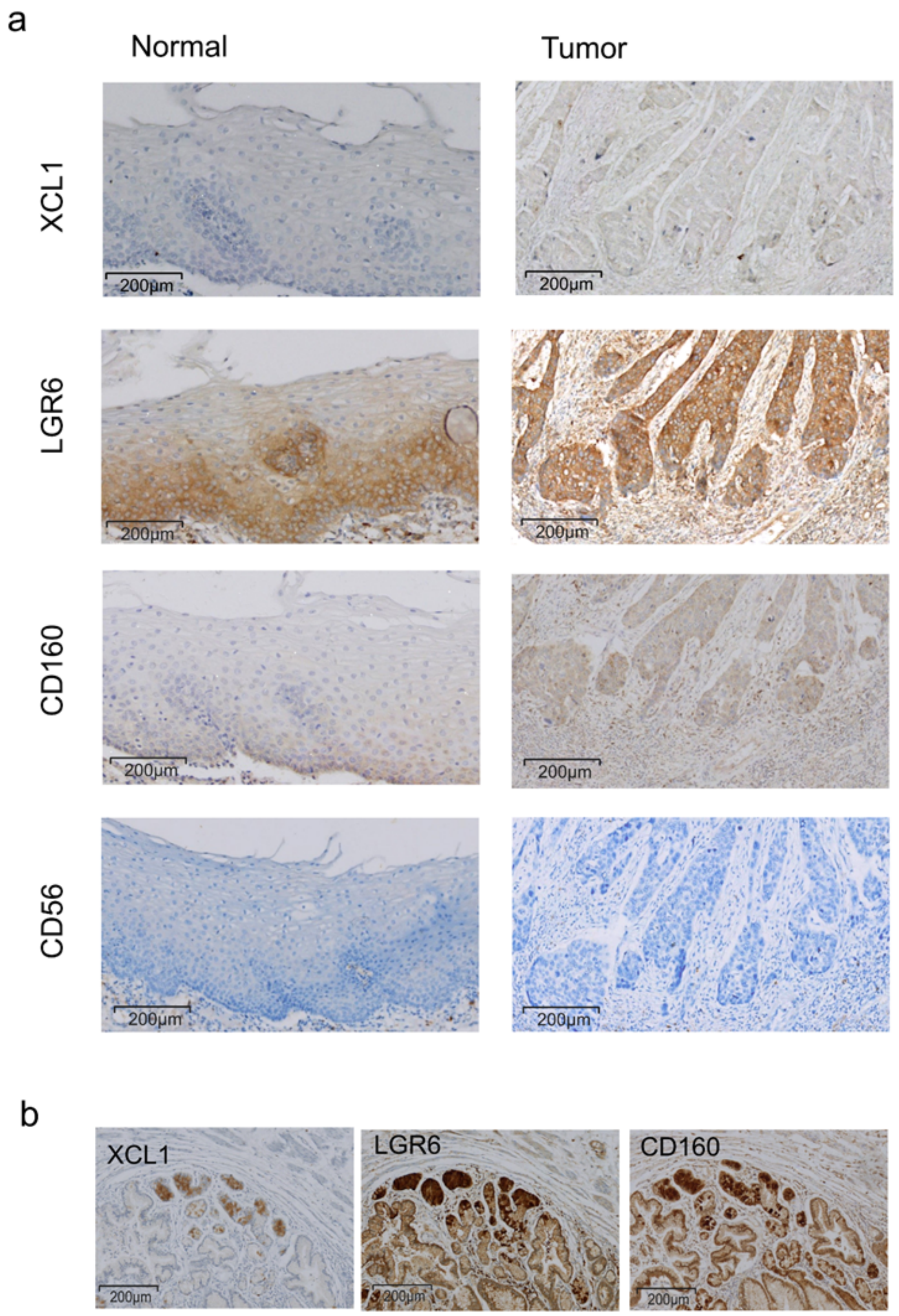

Supplementary Figure 12. Elevated expression of XCL1 is associated with worse survival. ESCC tumour samples showed higher expression of XCL1 (p = 3.33e-11) (a) and XCL2(p =2.2e-16)(b) compared to the adjacent matched normal. Two-sided Wilcoxon rank sum test was used to determine the differences between groups, The whiskers extending to a maximum of 1.5 times the interquartile range beyond the box. c, Survival analysis showed that high XCL1 expression is negatively associated with overall survival. d, The scatter plot in gene expression log2(RPKM+1) between XCL1 and LGR6 for 22 ESCC lines is shown. Source data are provided as a Source Data file.

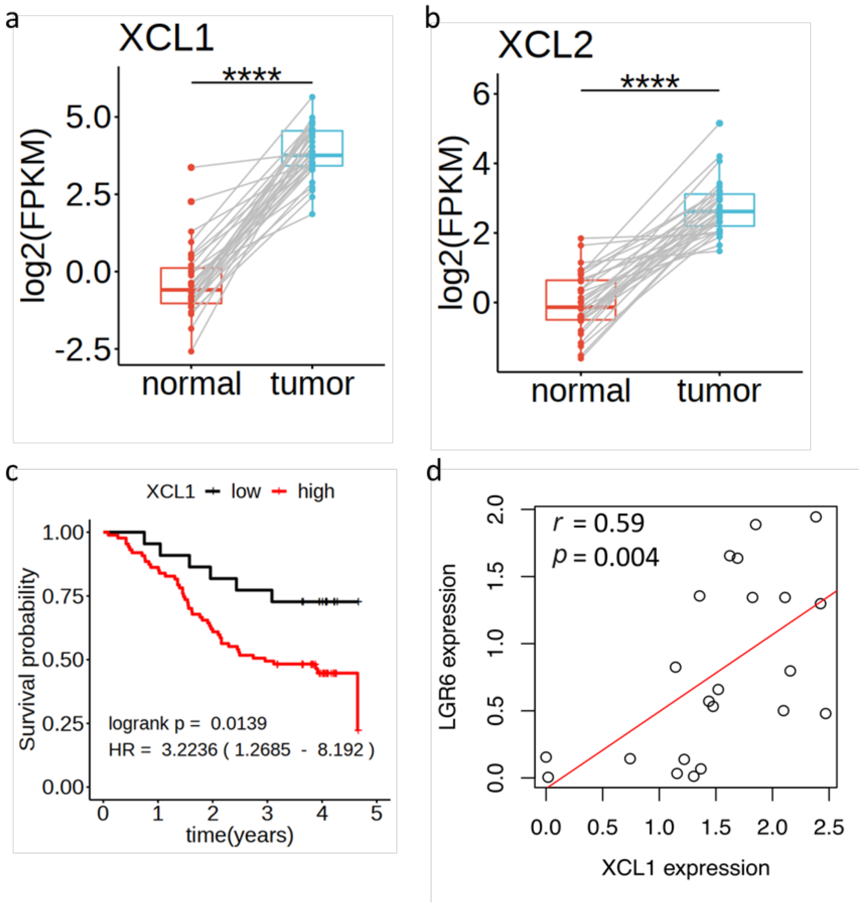

Supplementary Fig. 13. Comparison of drug metabolism of cytochrome P450 and retinol metabolism gene activities between XCL1-high ESCC cells and the metabolic subtype. Scatter plots of the t-statistics from the differential expression analysis between XCL1 high vs. low cells and metabolic vs. other subtypes were shown for genes involved in (a) drug metabolism of cytochrome P450 and (b) retinol metabolism. Correlation coefficient and associated p-value were shown in the plots. c, The overlap of significantly upregulated genes between XCL1-high and metabolic subtype showed very minimum number of signature genes shared by them.

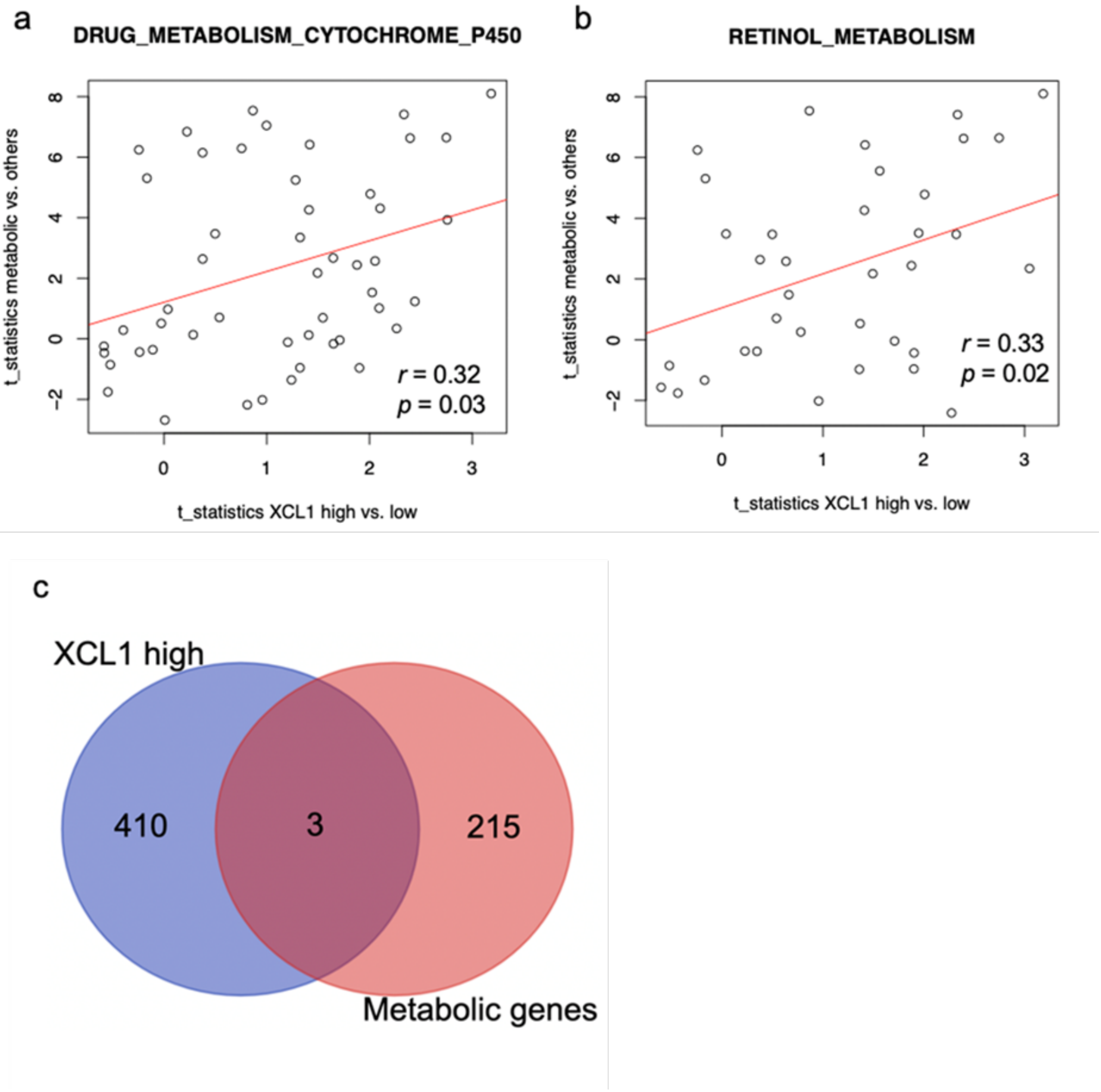

Supplementary Fig. 14. Cell cycle analysis of XCL1 overexpressed cell lines. a, overexpression of XCL1 in KYSE-180 and KYSE-410 cell lines. b, Cell cycle of XCL1 overexpressed and the control cell lines. EdU incorporation analysis for XCL1 overexpressed cells in comparison to control cells for (c) KYSE-410 and (d) KYSE-180. Source data are provided as a Source Data file.

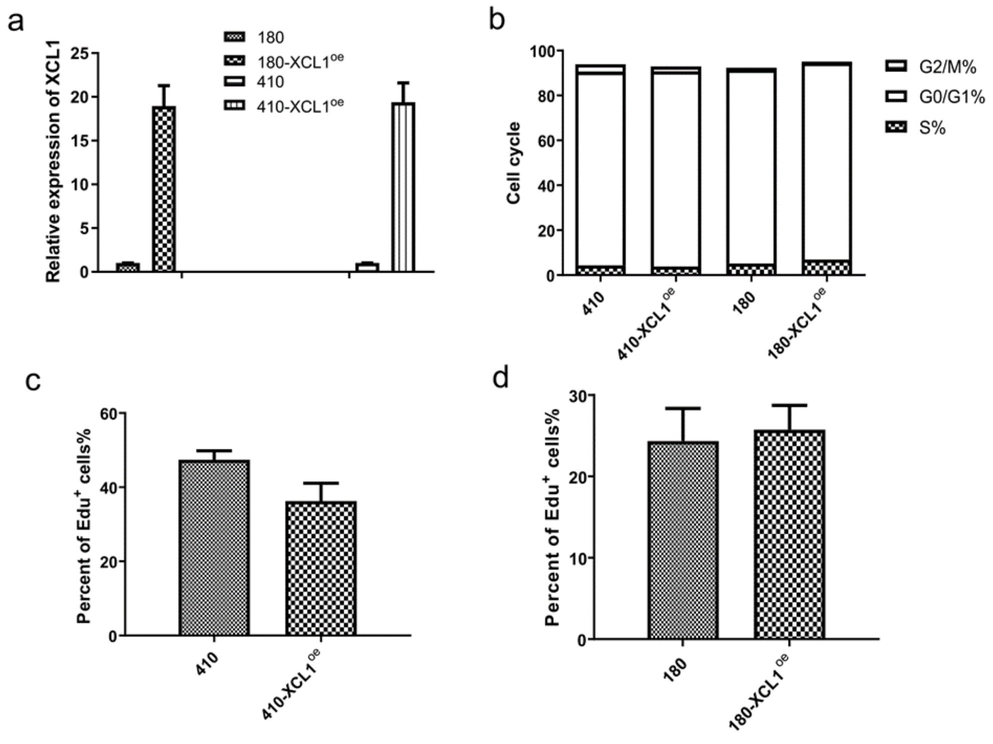

Supplementary Fig. 15. XCL1 expression in ESCC cell lines and other cancer tissues. a, Relative mRNA expression of XCL1 in ESCC cell lines using KYSE-150 cell as control by quantitative real-time-PCR (qRT-PCR). b, Relative XCL1 mRNA expression in XCL1 overexpressing KYSE-150 cells compared with control cells by qRT-PCR. Statistical significance was derived from two-sided Student t-test is shown, **** p<0.0001. c, The tissue images of XCL1 expression in tumour tissues of colorectal cancer, liver and renal cancer were shown, along with XCL1 survival plots in three cancer types demonstrating high XCL1 expression is associated with worse overall survival. All data were from Human Protein Atlas. d, Cell proliferation of XCL1 overexpressing and control ESCC cells using KYSE-150 and KYSE-450 lines. In a and b, data are presented as mean values + standard deviation. Source data are provided as a Source Data file.

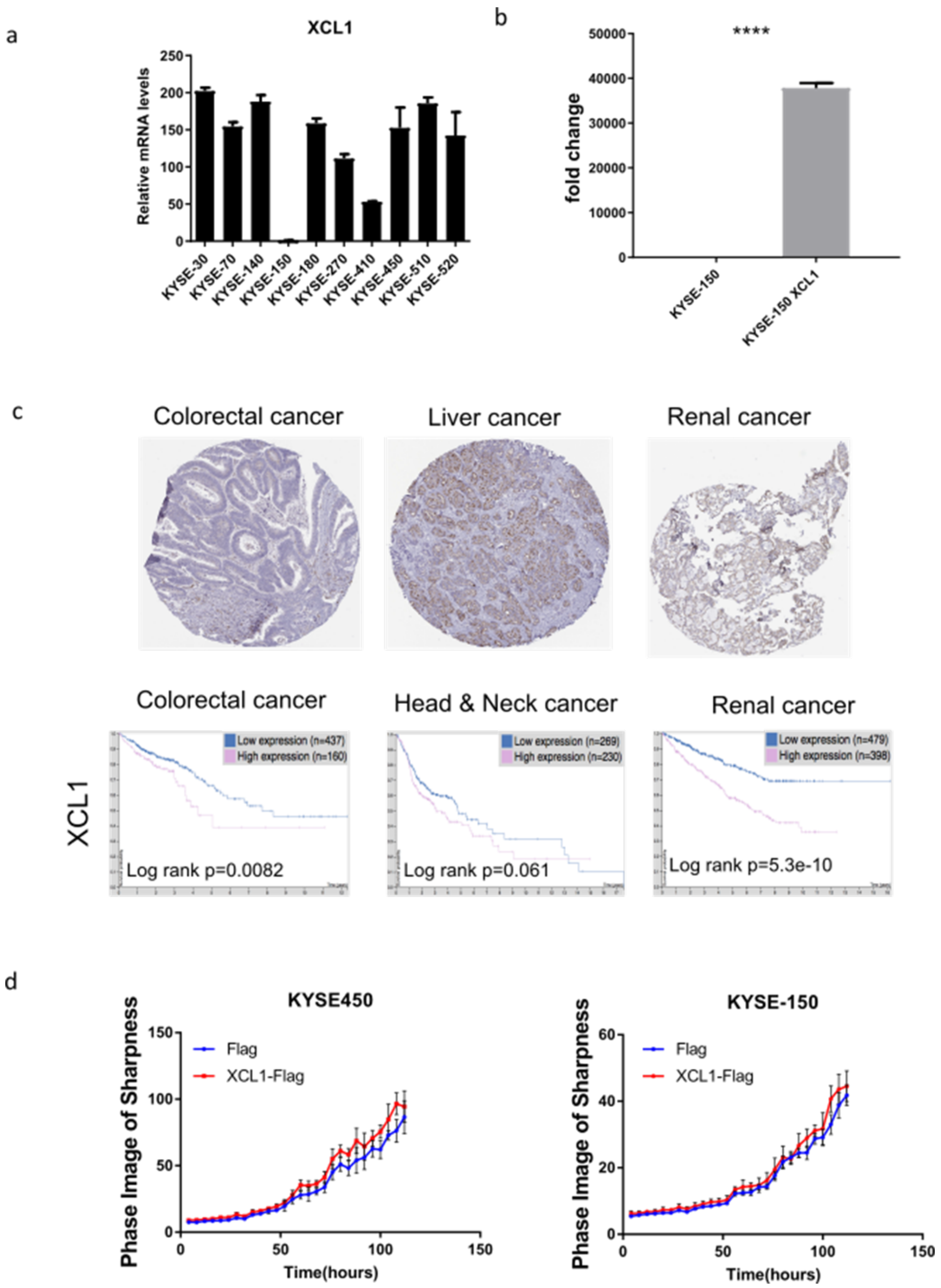

Supplementary Fig. 16. The genomic landscape of ESCC. a, There is no correlation between tumour purity and the number of mutations or subclonal mutations. **b**, The number of non-silent mutations and somatic copy number profiling were similar among four ESCC subtypes. the box bounds the interquartile range divided by the median, with the whiskers extending to a maximum of 1.5 times the interquartile range beyond the box. **c,** 10 ESCC driver genes were identified by at least two driver gene methods. **d**, The proportion of samples with previously reported ESCC subtype specific alterations among our four transcriptomic subtypes. Source data are provided as a Source Data file.

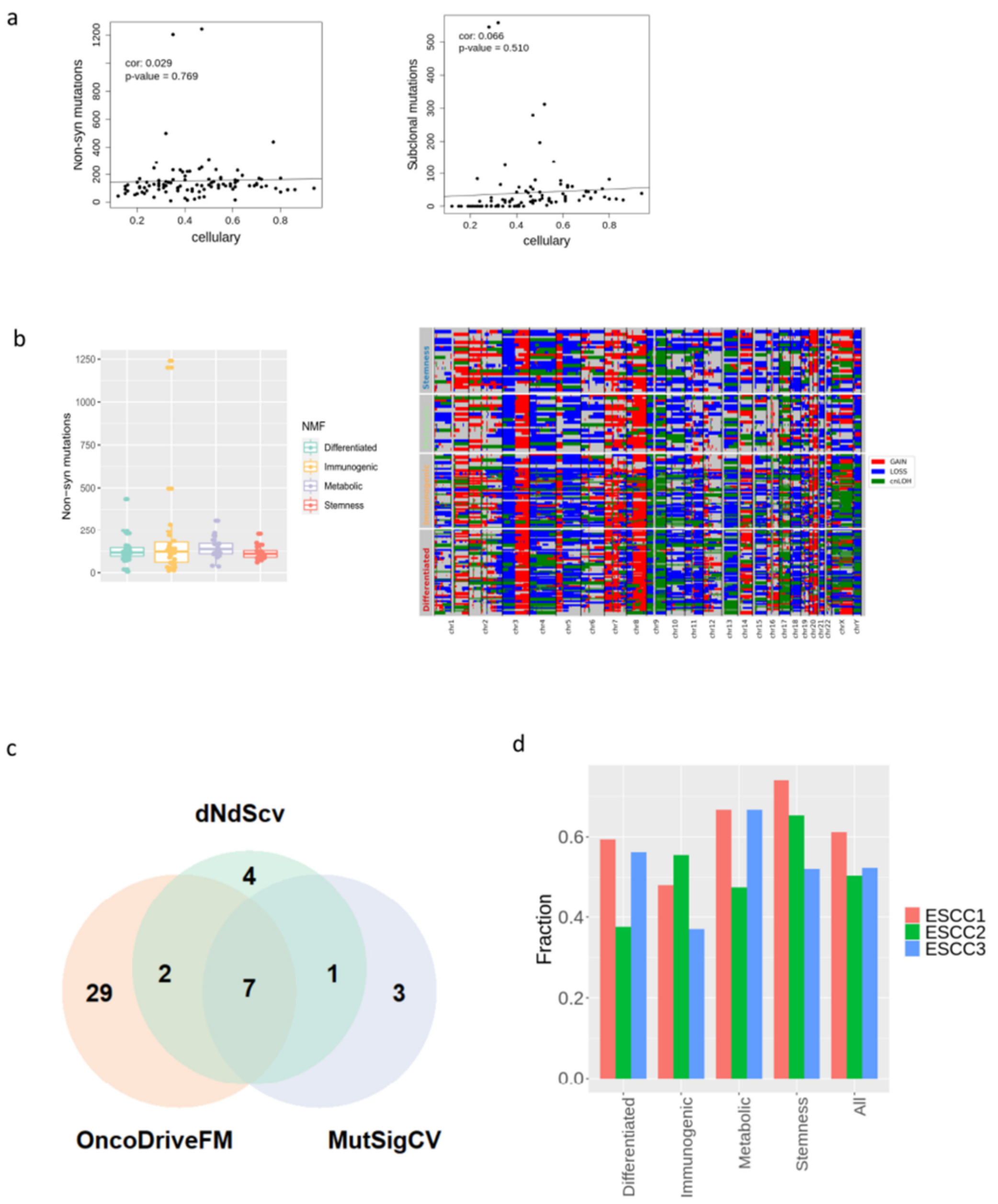

Supplementary Fig. 17. *EP300* overexpression and mutations in ESCC. a, The representative gene sets of immunogenic and metabolic subtypes, 'immunogenic_up' and 'metabolic_up' were downregulated in *EP300*-mutated samples, and 'differentiated_up' gene set was also massively downregulated in *EP300*-high expression samples. **b**, Gene expression of *EP300* among four transcriptomic subtypes. The whiskers extending to a maximum of 1.5 times the interquartile range beyond the box. **c**, The scatter plot and correlations of gene expression between NK marker genes *XCL1/2*, *CD160* and *EP300*. Source data are provided as a Source Data file.

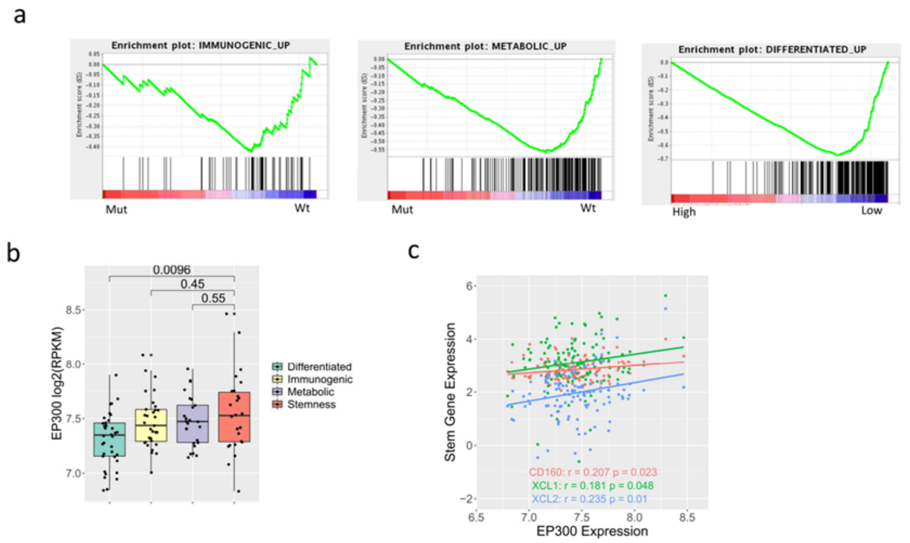

Supplementary Fig. 18. Functional mutation enrichment and pathway activity of cancer hallmark gene sets. a, Functional mutation enrichment scores for four gene sets where significant differences were identified among the four subtypes. the Kruskal–Wallis test was used for the comparisons. *, *p*<0.05; **, *p*<0.01. The whiskers extending to a maximum of 1.5 times the interquartile range beyond the box. **b,** Heatmap of gene sets where significant differences in GSVA pathway activity scores were identified among subtypes (the Kruskal–Wallis test, $p < 0.01$). **c,** Fraction of non-silent mutations, and functional prediction of missense mutations in the Wnt singaling are shown among the four subtypes.

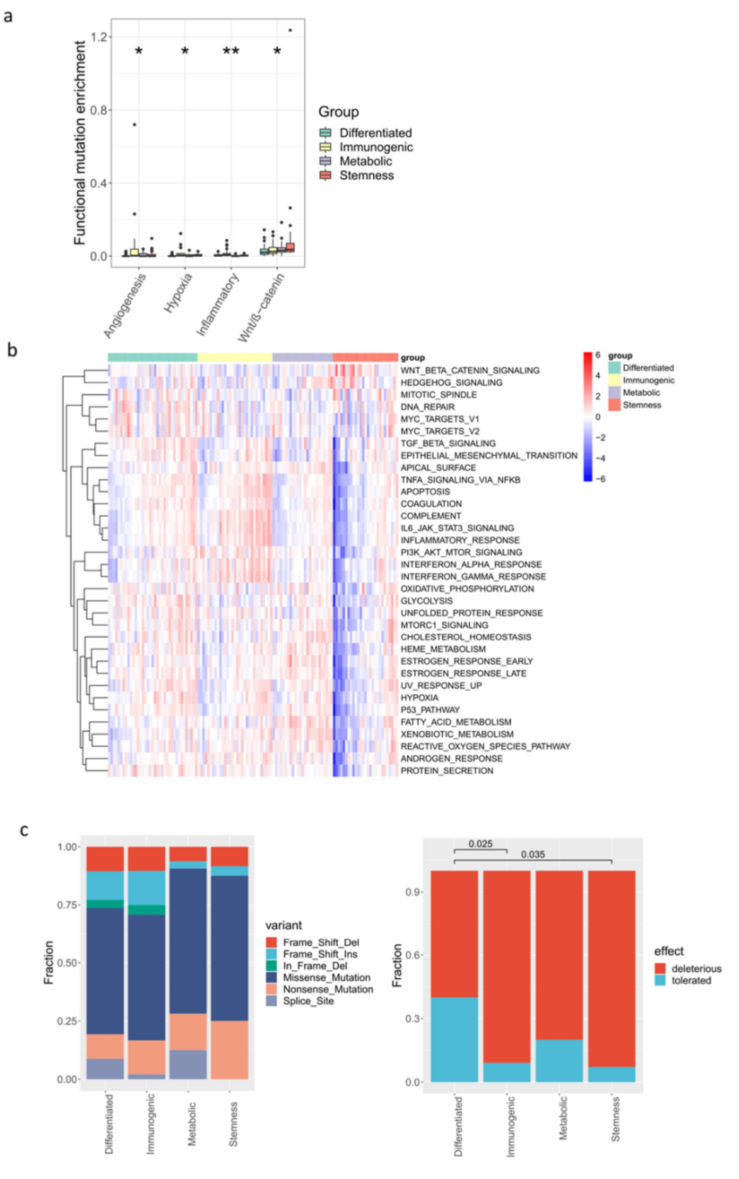

Supplement: Supplementary file 1 — Supplementary Information [file 41467_2024_53164_MOESM1_ESM.pdf]
