# Supplementary material for: The integrated molecular and histological analysis defines subtypes of esophageal squamous cell carcinoma
Source: Nat Commun. 2024 Oct 18;15:8988. doi: 10.1038/s41467-024-53164-x (PMC11487165; doi:10.1038/s41467-024-53164-x)
Supplement: Supplementary file 3 — Description of Additional Supplementary Files [file 41467_2024_53164_MOESM3_ESM.pdf]

Description of Additional Supplementary Files

Supplementary Data 1. Clinical information of 120 ESCC patients in our cohort.

Supplementary Data 2. Representative genes from the four transcriptomic subtypes. Differential expression analysis was performed for each cluster versus all other samples using limma.

Supplementary Data 3. Gene set enrichment analysis of representative genes in each subtype against the canonical pathways of the mSigDB database using a hypergeometric test. For each cluster, the significantly upregulated genes against all other samples (adjuste $p < 0.05$) were used for the hypergeometric test for the enrichment. Top significantly enriched pathways were selected for each cluster, FDR < 0.1. For the stemness subtype, due to the smaller gene set and also many genes being noncoding genes, p values in the hypergemetric test could not be adjusted

Supplementary Data 4. Top five histopathological imaging features of each transcriptomic group. Feature names were shown based on their corresponding CNN models.

Supplementary Data 5. Comparisons of histopathological Meta Features among ESCC transcriptome subtypes. The Wilcox Rank-Sum test was used.

Supplementary Data 6. The expression of LGR6,XCL1 and CD160 in esophageal squamous cell carcinoma determined by IHC staining

Supplementary Data 7. The proportion of XCL1 and CD160 expression, co-stained with LGR6 in the serial sections of the same samples in esophageal squamous cell carcinoma determined by IHC staining.

Supplementary Data 8. Significantly differentially expressed between XCL1 high vs low esophageal SCC cell lines (22 in total from CCLE), raw $p < 0.01$.

Supplementary Data 9. The drug screening of XCL1 high and low ESCC cell lines extracted from the Genomics of Drug Sensitivity in Cancer (GDSC) resource. The z-transformed IC50 scores were extracted. The GDSC1 data set was used. T-test was further performed between XCL1 high vs. low samples based on the z-transfored IC50 values.

Supplementary Data 10. Summary data of the 103 whole exome sequencing samples.

Supplementary Data 11. The EP300 mutation details for 8 ESCC samples.
