# Supplementary material for: The integrated molecular and histological analysis defines subtypes of esophageal squamous cell carcinoma
Source: Nat Commun. 2024 Oct 18;15:8988. doi: 10.1038/s41467-024-53164-x (PMC11487165; doi:10.1038/s41467-024-53164-x)
Supplement: Supplementary file 15 — Reporting Summary [file 41467_2024_53164_MOESM15_ESM.pdf]

nature portfolio

Corresponding author(s): DAPR
Manuscript: NCOMMS-21-30906C
Wencai Li
Jun Wang
Yaohe Wang

Last updated by author(s): Jun 10, 2024

Reporting Summary

Nature Portfolio wishes to improve the reproducibility of the work that we publish. This form provides structure for consistency and transparency in reporting. For further information on Nature Portfolio policies, see our Editorial Policies and the Editorial Policy Checklist.

Statistics

For all statistical analyses, confirm that the following items are present in the figure legend, table legend, main text, or Methods section.

n/a	Confirmed	
☐	☒	The exact sample size (*n*) for each experimental group/condition, given as a discrete number and unit of measurement
☐	☒	A statement on whether measurements were taken from distinct samples or whether the same sample was measured repeatedly
☐	☒	The statistical test(s) used AND whether they are one- or two-sided<br>*Only common tests should be described solely by name; describe more complex techniques in the Methods section.*
☐	☒	A description of all covariates tested
☐	☒	A description of any assumptions or corrections, such as tests of normality and adjustment for multiple comparisons
☐	☒	A full description of the statistical parameters including central tendency (e.g. means) or other basic estimates (e.g. regression coefficient) AND variation (e.g. standard deviation) or associated estimates of uncertainty (e.g. confidence intervals)
☐	☒	For null hypothesis testing, the test statistic (e.g. *F*, *t*, *r*) with confidence intervals, effect sizes, degrees of freedom and *P* value noted<br>*Give P values as exact values whenever suitable.*
☒	☐	For Bayesian analysis, information on the choice of priors and Markov chain Monte Carlo settings
☐	☒	For hierarchical and complex designs, identification of the appropriate level for tests and full reporting of outcomes
☐	☒	Estimates of effect sizes (e.g. Cohen's *d*, Pearson's *r*), indicating how they were calculated

Our web collection on statistics for biologists contains articles on many of the points above.

Software and code

Policy information about availability of computer code

Data collection

FastQC(0.11.7)
SOAPnuke1.5.6
Salmon(version 0.9.0)
'tximport' R package
Gene Set Enrichment Analysis (GSEA)
'NMFConsensus' algorithm in GenePattern
Consenseclusterplus Version 1.35.0
Limma R package bioconductor
mSigDB database (v.6.2)
BWA(0.7.17)
GATK4(4.0.6.0)
mutect2 and strelka2 (2.8.4)
Sequenza(2.1.2)
PyClone 0.12.8
GSVA R package (1.42)
Cancer Cell Line Encyclopedia (CCLE) data portal (https://portals.broadinstitute.org/ccle)
Deep learning models, Inception-V3, Inception-ResNet-V2, DenseNet-121, VGG16 and ResNet-50
Seurat R package
The Genomics of Drug Sensitivity in Cancer (GDSC) data repository (https://www.cancerrxgene.org/downloads/bulk_download).

	The UCSC Xena Browser, TCGA Esophageal Cancer (ESCA) (n = 198)<br>R survminer 0.4.3 package
Data analysis	Bioinformatic tools used in this study have been mentioned in methods or results section. R and python scripts and packages used to analyze the data under conda environments. All packages mentioned are freely available online.

For manuscripts utilizing custom algorithms or software that are central to the research but not yet described in published literature, software must be made available to editors and reviewers. We strongly encourage code deposition in a community repository (e.g. GitHub). See the Nature Portfolio guidelines for submitting code & software for further information.

Data

Policy information about availability of data

All manuscripts must include a data availability statement. This statement should provide the following information, where applicable:

- Accession codes, unique identifiers, or web links for publicly available datasets
- A description of any restrictions on data availability
- For clinical datasets or third party data, please ensure that the statement adheres to our policy

Sequence data used during the study has been deposited at National Genomics Data Centre of China (https://bigd.big.ac.cn/) and the Bioproject Access ID is PRJCA001577 [https://ngdc.cncb.ac.cn/bioproject/browse/PRJCA001577]. This BioProject has two associated GSA accession numbers: HRA000111 [https://ngdc.cncb.ac.cn/gsa-human/browse/HRA000111] hosts the raw sequencing data of RNA-seq, while HRA000112 [https://ngdc.cncb.ac.cn/gsa-human/browse/HRA000112] has the raw sequencing data of whole exome sequencing. The availability of the data has been approved by the Human Genetic Resources Registration System of the Ministry of Science and Technology of People's Republic of China with the registration number of 2024BAT00864. Single cell RNA-seq data were downloaded from Gene Expression Omnibus under the accession number of GSE160269.

The data associated with PRJCA001577, i.e., GSA accession number HRA000111 and HRA000112, was under controlled access. Users can applied for the access to the data through the National Genomics Data Centre of China GSA system with the permission approved by the Data Access Committee(DAC) HDAC000064 [https://ngdc.cncb.ac.cn/gsa-human/browse/dac/HDAC000064].

All the codes that were used for analyses and the generation of figures are available at https://github.com/Zhong2020/ESCCproject. All the codes used for the deep-learning analysis are available at https://github.com/BioInforCore-BCI/giExtract. (doi: 10.5281/zenodo.11049708)

Three publicly available gene expression data sets from GEO were used : GSE53625 [https://www.ncbi.nlm.nih.gov/geo/query/acc.cgi?acc=GSE53625],GSE47404 [https://www.ncbi.nlm.nih.gov/geo/query/acc.cgi?acc=GSE47404], GSE160269 [https://www.ncbi.nlm.nih.gov/geo/query/acc.cgi?acc=GSE160269]. TCGA Esophageal Cancer (ESCA) RNA-seq data was also used [https://xenabrowser.net/datapages/?dataset=TCGA.ESCA.sampleMap%2FHiSeq&host=https%3A%2F%2Ftcga.xenahubs.net&removeHub=https%3A%2F%2Fxena.treehouse.gi.ucsc.edu%3A443], and 90 ESCC samples were further extracted.

Research involving human participants, their data, or biological material

Policy information about studies with human participants or human data. See also policy information about sex, gender (identity/presentation), and sexual orientation and race, ethnicity and racism.

Reporting on sex and gender	Both male and female patients are included in this study, all 120 patients signed the consent and the study is approved by the ethic committee of Zhengzhou University. This is also the case for the validation cohort of 65 ESCC patients.<br>No sex/gender or age specific analysis was carried out, as these were not associated with our molecular signatures.
Reporting on race, ethnicity, or other socially relevant groupings	All patients were from a high ESCC incidence region of Henan Province in China. Thus, race, ethnicity, or other socially relevant groupings were not considered.
Population characteristics	120 patients diagnosed with esophageal squamous cell carcinoma from 2013 to 2016. None of these patients received any radiotherapy or chemotherapy before surgery. the clinicopathological information can be found in supplementary table1.<br>A validation cohort of 65 ESCC patient samples were also identified from Anyang Cancer Hospital, all primary treatment naïve tumours. All patients were informed and signed a patient informed consent, and the study was approved by the Ethics Committee of Zhengzhou University.
Recruitment	Each participating institution provided samples from existing banked tissues with appropriate permissions for secondary research use.
Ethics oversight	This research complies with all relevant ethical regulations and was approved by the Ethics Committee of Zhengzhou University. The patient samples were enrolled from Anyang Cancer Hospital under the approve of ethics committee of Both Anyang Cancer Hospital and The First Affiliated Hospital of Zhengzhou University.

Note that full information on the approval of the study protocol must also be provided in the manuscript.

Field-specific reporting

Please select the one below that is the best fit for your research. If you are not sure, read the appropriate sections before making your selection.

☒ Life sciences ☐ Behavioural & social sciences ☐ Ecological, evolutionary & environmental sciences

For a reference copy of the document with all sections, see nature.com/documents/nr-reporting-summary-flat.pdf

Life sciences study design

All studies must disclose on these points even when the disclosure is negative.

Sample size	Comprehensive genomic-transcriptomic characterizations and AI-aided histopathological image analysis of 120 Chinese ESCC patients were performed. No sample size calculation was performed, as this n=120 cohort still represents the largest treatment naive primary cohort to date with detailed clinical follow-up. Our main aim was to identify molecular and histological heterogeneity in ESCC, thus the more samples the better. And n=120 is the highest number we could archive when the project started.<br><br>Expression for 22 esophageal SCC cancer cell lines were extracted. XCL1 high and low groups are based on hierarchical clustering of profiled genes. n=22 ESCC cell lines were all ESCC lines profiled in CCLE.<br>For SFRP1 overexpression and knockdown analysis, n=6 replicates in each group was used.<br>In the IC50 experiment, for each cell line we conducted three repeated experiments to determine the IC50.<br>For the single cell RNA-seq data, data for ~200k cells from 60 individuals were extracted and used.<br>For XCL1 and LGR6, IHC was performed for 98 samples; while for CD160 and LGR6 co-staining, 90 samples were sufficiently profiled.<br>For the digital pathology study, whole slide images of 91 patients were used.
Data exclusions	For the whole exome sequencing samples, 17 of the 120 samples were excluded for WES sequencing because of the low qualify of DNA.<br>For the digital pathology study, each WSI was manually reviewed by a qualified pathologist, and poor-quality images were discarded under the direct pathologist's supervision. The poor quality of imaging means, the sections were folded without clear morphology or there were not enough tumour cells presented in the slides obtained from the department of histopathology. Only those images with tumours and free of technical artefacts were used for further analysis. A total of 91 WSIs were retained for the deep-learning analysis, i.e., differentiated group, n=28; immunogenic, n=27; metabolic, n=18; stemness, n=18.
Replication	Replications are indicated in each figure and corresponding figure legends. Also see "sample size" above
Randomization	Randomization was not applicable to the study, as this is not a clinical trial. Eligible patients were identified using our clinical database, and their samples were used for the molecular and histological profiling.
Blinding	Blinding was not applicable to the study, as this is not a clinical trial but biomarker discovery.

Reporting for specific materials, systems and methods

We require information from authors about some types of materials, experimental systems and methods used in many studies. Here, indicate whether each material, system or method listed is relevant to your study. If you are not sure if a list item applies to your research, read the appropriate section before selecting a response.

Materials & experimental systems

n/a	Involved in the study	
☐	☒	Antibodies
☐	☒	Eukaryotic cell lines
☒	☐	Palaeontology and archaeology
☐	☒	Animals and other organisms
☐	☒	Clinical data
☒	☐	Dual use research of concern
☒	☐	Plants

Methods

n/a	Involved in the study	
☒	☐	ChIP-seq
☒	☐	Flow cytometry
☒	☐	MRI-based neuroimaging

Antibodies

Antibodies used	anti-SFRP1 (1:500, Atlas antibodies,HPA064870), anti-GAPDH (1:5000, ProteinTech, 60004-1-Ig), HRP-Goat Anti-Rabbit IgG (1:5000, ZSBIO, ZB-5301), HRP-Goat anti-mouse IgG (1:5000, ZSBIO, ZB-5305),anti-XCL1 (1:400, Atlas antibodies,HPA057725), anti-LGR6 (1:100, Abcam,126747), anti-CD160 (1:300, Origene, TA349762), anti-CD8 (Genetech, GT211207), anti-CD4 (Maixin Biotechnology).
Validation	SFRP1 and XCL1 antibodies are selected from Human Protein Atlas (https://www.proteinatlas.org/ENSG00000104332-SFRP1/summary/antibody,https://www.proteinatlas.org/ENSG00000143184-XCL1/summary/antibody). GAPDH Monoclonal antibody is widely used and validated (https://www.ptglab.com/products/GAPDH-Antibody-60004-1-Ig.htm). HRP-Goat Anti-Rabbit and anti-mouse IgG, CD160 ,CD8 and CD4 antibodies are widely used in China pathology laboratories and validated in this study with controls.<br><br>For anti-CD160, CD160 Mouse Monoclonal Antibody [Clone ID: OTI4C12], a validated antibody by Origene was used (https://www.origene.com/catalog/antibodies/primary-antibodies/ta809702/cd160-mouse-monoclonal-antibody-clone-id-oti4c12)<br>For anti-LGR6, rabbit Recombinant Monoclonal GPCR LGR6 antibody was used, https://www.abcam.com/en-gb/products/primary-antibodies/gpcr-lgr6-antibody-epr6874-ab126747

Eukaryotic cell lines

Policy information about cell lines and Sex and Gender in Research

Cell line source(s)	The cell lines KYSE-30, KYSE-70, KYSE-140, KYSE-150, KYSE-180, KYSE-270, KYSE-410, KYSE-450, KYSE-510 and KYSE-520 were obtained from the Japanese Collection of Research Bioresources Cell Bank (JCRB, Osaka, Japan). HEK-293T was purchased from the Cell Bank of the Type Culture Collection Committee of the Chinese Academy of Sciences (Shanghai, China).
Authentication	All cell lines used in this study were authenticated by supplier using STR profiling
Mycoplasma contamination	All cell lines were confirmed negative for mycoplasma contamination.
Commonly misidentified lines (See ICLAC register)	No misidentified cell lines were used in this study.

Animals and other research organisms

Policy information about studies involving animals; ARRIVE guidelines recommended for reporting animal research, and Sex and Gender in Research

Laboratory animals	-5 weeks old female BALB/c Nude mice were purchased from Beijing Vital River Laboratory (Beijing, Cat# 401).
Wild animals	The study did not involve wild animals.
Reporting on sex	Sex was not considered in this study. Only female mice were used.
Field-collected samples	No field collected samples were used in this study.<br>All animal assay were conducted in accordance with accordance with the Guide for the Care and Use of Laboratory Animals. Mice were executed and xenografts volume were measured after 30 days.
Ethics oversight	The animal assays were approved by the Animal Welfare and Research Ethics Committee of Zhengzhou University (Zhengzhou, China).

Note that full information on the approval of the study protocol must also be provided in the manuscript.

Clinical data

Policy information about clinical studies

All manuscripts should comply with the ICMJE guidelines for publication of clinical research and a completed CONSORT checklist must be included with all submissions.

Clinical trial registration	Not applicable
Study protocol	120 patients diagnosed with esophageal squamous cell carcinoma from 2013 to 2016 were enrolled from Anyang Cancer Hospital under the approve of ethics committee of Both Anyang Cancer Hospital and The First Affiliated Hospital of Zhengzhou University. None of these patients received any radiotherapy or chemotherapy before surgery and pathology diagnosis were confirmed by three independent pathologists. Tumour samples and adjacent normal tissues at least 5 cm away from paired tumour tissues were collected and placed in liquid nitrogen within 30 minutes after surgery operation. A validation cohort of 65 ESCC patient samples were also identified from Anyang Cancer Hospital, all primary treatment naïve tumours. All patients were informed and signed a patient informed consent, and the study was approved by the Ethics Committee of Zhengzhou University.
Data collection	Tumour samples and adjacent normal tissues at least 5 cm away from paired tumour tissues were collected and placed in liquid nitrogen within 30 minutes after surgery operation. Long term follow up data (more than four years follow up after surgical resections) was carefully collected by the clinical team.
Outcomes	In the Kaplan Meier analysis, the overall survival was the primary clinical outcome.

Plants

Seed stocks	Not applicable
Novel plant genotypes	Not applicable
Authentication	Not applicable
